# Screening effects of HCN channel blockers on sleep/wake behavior in zebrafish

**DOI:** 10.3389/fnins.2024.1375484

**Published:** 2024-03-19

**Authors:** Fusun Doldur-Balli, Sandra P. Smieszek, Brendan T. Keenan, Amber J. Zimmerman, Olivia J. Veatch, Christos M. Polymeropoulos, Gunther Birznieks, Mihael H. Polymeropoulos

**Affiliations:** ^1^Division of Sleep Medicine, Department of Medicine, Perelman School of Medicine, University of Pennsylvania, Philadelphia, PA, United States; ^2^Vanda Pharmaceuticals Inc., Pennsylvania, Washington, DC, United States; ^3^Department of Psychiatry and Behavioral Sciences, University of Kansas Medical Center, Kansas City, KS, United States

**Keywords:** drug screening, sleep/wake, zebrafish, Ivabradine (Corlanor), Zatebradine hydrochloride, ZD7288, HCN channel blocker

## Abstract

Hyperpolarization-activated cyclic nucleotide-gated (HCN) ion channels generate electrical rhythmicity in various tissues although primarily heart, retina and brain. The HCN channel blocker compound, Ivabradine (Corlanor), is approved by the US Food and Drug Administration (FDA) as a medication to lower heart rate by blocking hyperpolarization activated inward current in the sinoatrial node. In addition, a growing body of evidence suggests a role for HCN channels in regulation of sleep/wake behavior. Zebrafish larvae are ideal model organisms for high throughput drug screening, drug repurposing and behavioral phenotyping studies. We leveraged this model system to investigate effects of three HCN channel blockers (Ivabradine, Zatebradine Hydrochloride and ZD7288) at multiple doses on sleep/wake behavior in wild type zebrafish. Results of interest included shorter latency to daytime sleep at 0.1 μM dose of Ivabradine (ANOVA, p: 0.02), moderate reduction in average activity at 30 μM dose of Zatebradine Hydrochloride (ANOVA, p: 0.024) in daytime, and increased nighttime sleep at 4.5 μM dose of ZD7288 (ANOVA, p: 0.036). Taken together, shorter latency to daytime sleep, decrease in daytime activity and increased nighttime sleep indicate that different HCN channel antagonists affected different parameters of sleep and activity.

## 1 Introduction

Hyperpolarization-activated cyclic nucleotide-gated (HCN) ion channels are members of the family of the voltage gated ion channels (Sartiani et al., 2017). HCN channels are encoded by the *HCN1-4* gene family (Chang et al., 2019) and can form homotetramers or heterotetramers with specific biophysical properties (Sartiani et al., 2017). These integral membrane proteins (Flynn and Zagotta, 2018) generate an inward current in heart (I_f_) and nerve cells (I_h_) (Novella Romanelli et al., 2016). HCN channels are known as pacemakers (Wobig et al., 2020); they modulate cardiac rhythmicity and neuronal excitability (Wobig et al., 2020). Functions of HCN channels in photoreceptors include adaptation of the vertebrate retina to visual stimuli (Barrow and Wu, 2009). Notably, HCN channels are also involved in regulation of sleep/wake behavior (Lewis and Chetkovich, 2011; Sartiani et al., 2017; Byczkowicz et al., 2019; Chang et al., 2019) by contributing to the formation of spindle waves (McCormick and Pape, 1990; Bal and McCormick, 1996) and slow wave oscillations during non-Rapid Eye Movement (NREM) sleep (Kanyshkova et al., 2009; Zobeiri et al., 2018). There are different reports on how HCN channels fulfill sleep related functions. One line of research suggests that inhibition of HCN channels, thereby inhibition of I_h_ current, via local infusion of melatonin in mouse lateral hypothalamus is associated with reductions in wakefulness (Huang et al., 2020). In contrast, inhibition of I_h_ current via orexin A application to mouse prelimbic cortex increased wakefulness (Li et al., 2010). Another study reported sleep fragmentation in a *Drosophila* mutant model, which lacks I_h_ current; however, no significant difference in total sleep amount was noted between mutant and control flies (Gonzalo-Gomez et al., 2012). These different findings reported in the literature led us to test effects of HCN channel blocker compounds on rest/wake behavior in zebrafish as they are a diurnal vertebrate system for performing high-throughput screening of small molecule compounds. We evaluated Ivabradine (Corlanor), Zatebradine hydrochloride and ZD7288 in this study. Specifically, Ivabradine has been observed to inhibit inward current in cell lines originated from human embryonic kidney cells and Chinese hamster ovary cells and rabbit sinoatrial nodes (Novella Romanelli et al., 2016). Zatebradine inhibited inward current in human embryonic kidney cell lines and Xenopus oocytes (Novella Romanelli et al., 2016). Administration of ZD7288 was found to inhibit inward current in human embryonic kidney cell lines, Chinese hamster ovary cell lines, Xenopus oocytes, rat dorsal ganglion neurons, spontaneously hypertensive ventricular myocytes and Guinea pig sinoatrial nodes (Novella Romanelli et al., 2016). These compounds block HCN subunits nonselectively (Novella Romanelli et al., 2016; Zhong and Darmani, 2021). All three compounds are pharmacological tools used to reduce heart rate (Novella Romanelli et al., 2016); however, Ivabradine is the only FDA approved drug used in patients with heart failure (Novella Romanelli et al., 2016). Drug screening studies using zebrafish models have been instrumental in detecting effects of small molecule compounds on regulation of sleep/wake behavior and circadian rhythm (Rihel et al., 2010a; Mosser et al., 2019). In addition, zebrafish can be utilized to identify mechanism of action of drugs (Rihel et al., 2010a; Hoffman et al., 2016; Mosser et al., 2019). The zebrafish model has several additional advantages, such as yielding a high number of offspring per breeding and high throughput assessment of sleep/wake (Oikonomou and Prober, 2017). Sleep phases and regulation of sleep in zebrafish are conserved and meet all the behavioral criteria that are used to define a sleep state (Zhdanova, 2006; Rihel et al., 2010b). Given these advantages, to reveal effects of HCN channel blocker compounds on sleep/wake behavior, we tested if wild type zebrafish larvae exposed to three compounds, administered at different dosages, expressed differences in multiple sleep-related traits when compared to vehicle (DMSO) exposed fish.

## 2 Materials and methods

### 2.1 Zebrafish sleep/wake assay

Larval zebrafish were raised on a 14 h light and 10 h dark cycle at 28.5°C. The entrainment and activity measurement equipment (ViewPoint Life Sciences Inc., aka Zebraboxes) houses 96 well plates and utilizes infrared lights to collect data. Data was collected every 60 s in quantization mode. Software was set for the following values: detection threshold: 20, burst: 29, and freeze: 3 (Doldur-Balli et al., 2023). White light is used to maintain day (lights on at 9: 00 am) and night (lights off at 11:00 pm) cycle. Recirculating water heated by temperature control unit (Corio CP BC4, Julabo GmbH) was utilized to ensure that zebrafish larvae were kept at optimum growth temperature (28.5°C) in the chamber of the equipment. Zebrafish larvae collected from a wild type line (AB line) were individually pipetted into each well of a 96 well plate (Whatman, catalog no: 7701–1,651) containing 650 μL of standard E3 embryo medium (5 mM NaCl, 0.17 mM KCl, 0.33 mM CaCl_2_, 0.33 mM MgSO_4_, pH 7.4) at 4 days post fertilization (dpf) (Lee et al., 2017, 2022). Embryo medium in the wells was topped off each morning once lights were on during experiment. Zebrafish experiments were performed in accordance with the University of Pennsylvania Institutional Animal Care and Use Committee guidelines.

### 2.2 Drug testing

**Figure 1:**
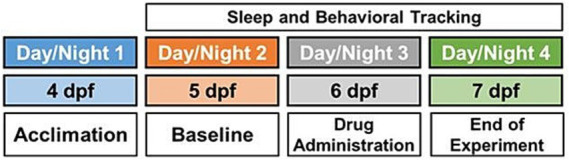
Schematic overview of experimental paradigm, including acclimation on experimental day 1 (4 days post fertilization [dpf]), baseline recording on day 2 (5 dpf), drug administration on day 3 (6 dpf) and sleep and behavioral tracking on days 2–4 (5–7 dpf).

Experiments were performed on 96 well plates. Four wells chosen at random (maximum one per row) did not include any larvae but were instead filled with standard embryo medium (E3 embryo medium) to serve as quality control (QC) for the settings, recording and sensitivity of the equipment. Ivabradine (Cayman, Cas Registry No. 148849–67-6), Zatebradine hydrochloride (Tocris, Cas Registry No. 91940–87-3) and ZD7288 (Tocris, Cas Registry No. 133059–99-1) were tested in this study. Each compound was tested at six concentrations varying between 0.1–30 μM (.i.e., 0.1 μM, 0.3 μM, 1.0 μM, 4.5 μM, 10 μM and 30 μM), as reported previously (Rihel et al., 2010a). Each drug was dissolved in DMSO. Stock solutions of Ivabradine, Zatebradine hydrochloride and ZD7288 were prepared at 35 millimolar, 40 millimolar and 30 millimolar concentrations, respectively. As indicated by the manufacturers; solubility of Ivabradine and Zatebradine hydrochloride in DMSO is 20 mg/mL and that of ZD7288 is 100 millimolar. Lower concentrations were obtained by serial dilution. Drug solutions were pipetted into the wells at the time of drug administration, thereby concentration of the stock solution was diluted 1,000 times in the wells (Rihel et al., 2010a). Each dose was tested on 11–12 larvae per plate, depending on the location of the randomly chosen QC wells, for evaluating the impact of different doses of the target drug on sleep and behavioral phenotypes. Zebrafish larvae were allowed to acclimate to the environment by spending the first night without any exposure to drugs and baseline sleep was observed during the second night. Drugs were then added at six days post fertilization at 5:00 pm; this was a one-time drug administration for all the tested doses and compounds in this study. 96 well plate was removed from the video monitoring equipment to administer drug compound and software continued to capture activity data. The peak in the sleep graph at the time of drug administration was formed when 96 well plate was removed from the equipment. Each assay was performed over a total of four days: acclimation on day 1, tracking baseline sleep on day 2, drug administration on day 3 and data acquisition between days 2–4 (see Figure 1). Concurrently, we studied 11–12 embryos that served as a DMSO (or drug vehicle, 1:1000 vol:vol) exposed control group and 11–12 embryos were exposed to 100 nM (0.1 micromolar) of melatonin as a positive control. Prior literature has utilized this concentration of melatonin to demonstrate sleep-promoting effects (Zhdanova et al., 2001), and our own proof-of-concept data shows that melatonin is very effective for increasing sleep in zebrafish larvae (see Supplementary Figure S1). The studies for Ivabradine were repeated six times (three replicates in two Zebraboxes) for a total of 66–72 fish for each drug concentration (11–12 fish per replicate) to ensure robust statistical power in the first drug screening assay. Based on statistical power analysis, providing an effect size of 0.8, appropriate sample size to determine significance was n = 25. Therefore, we concluded that three repeats of Zatebradine hydrochloride and ZD7288 assays using a different group of wild type embryos for each replicate would be sufficient by providing three biological replicates for a total of 33–36 fish for each drug concentration (11–12 fish per replicate, all replicates were carried out in the same Zebrabox for each drug).

### 2.3 Behavioral phenotyping

Data were analyzed using a custom designed MATLAB code (Lee et al., 2017, 2022). Behavioral tracking took place for 2 days starting from baseline sleep on day 5 of larval development (see Figure 1). The evaluated sleep phenotypes (see Supplementary Table S1) included total sleep duration, average activity, average waking activity, sleep bout numbers, consolidation of sleep (average sleep bout length) and latency to sleep as a measure relevant to insomnia (Doldur-Balli et al., 2023). Primary analyses were based on phenotypes calculated within the time window between 30 min after drug administration (drug administration was performed at 5:00 pm) and 11 pm (beginning of lights off period). Secondary analyses were performed for the night following drug administration (lights off period).

### 2.4 Statistical analysis

Analyses were performed to evaluate phenotypic effects of compounds of interest at six concentrations – 0.1 μM, 0.3 μM, 1.0 μM, 4.5 μM, 10 μM and 30 μM – as reported by Rihel et al. (2010a) using complementary approaches. First, to evaluate the relationship between drug doses and sleep phenotypes with minimal assumptions, we performed an analysis of variance (ANOVA) testing whether there were any differences in phenotypes among the experimental groups (DMSO and drug doses). If results for this overall ANOVA were significant (*p* < 0.05), we examined pairwise differences between drug doses and camera-matched DMSO controls to assess which groups were driving the overall differences, including calculation of standardized mean differences (SMDs). The standardized mean difference (SMD) was calculated by dividing the observed mean difference between groups by the pooled standard deviation. As defined by Cohen (Cohen, 1988), SMDs of 0.2, 0.5 and 0.8 represent small, medium and large differences, respectively. In addition to ANOVA, two complementary dose–response analyses were performed to evaluate whether a consistent change in sleep phenotypes was observed for increasing drug doses. First, we performed a linear trend analysis, including dose as an ordinal variable in the regression model (e.g., DMSO = 0, 0.1 μM = 1, 0.3 μM = 2, …, 30 μM = 6). This model treats differences between doses as similar in magnitude, asking whether there is a linear increase for higher dosage groups. Second, dose was included as a continuous variable in linear regression, to estimate the expected change in outcome for a 1 μM increase in drug dose; these analyses give increased weight to differences between DMSO and higher dosage groups (e.g., 10 μM or 30 μM). A *p*-value <0.05 was considered evidence of a significant association across all analyses. To maximize statistical power, analyses were performed pooling data from all experiments. To help account for potential batch effects, the experimental replicate (1, 2 or 3) was included as a covariate and analyses of Ivabradine also included a covariate for experimental box (1 or 2), as two different boxes were utilized. In addition, all analyses performed on data measured after drug administration were adjusted for baseline values of the given phenotype during the same time period prior (i.e., data from the day before and data from the night before were used as baseline values in primary and secondary analysis, respectively). Analyses in which significant associations in both ANOVA and dose–response analyses are observed were considered the most robust evidence for an effect of the drug compound. Results in which there were observed differences based on ANOVA but not following dose–response analyses were assumed to suggest a single dose of drug may be driving the overall results.

### 2.5 Power and sample size

Our study included between 33–36 larvae per drug concentration across three biological replicates. This represents nearly twice the maximum sample size utilized by a previous zebrafish drug screening study which detected significant effects (Rihel et al., 2010a). Furthermore, for pairwise contrasts, ≥33 animals per group were estimated to provide >80% power to detect standardized effect size differences (i.e., Cohen’s *d*) of at least 0.70 at an *α* = 0.05, which represent moderate-large effects. Analyses leveraging all data to examine the linear dose response (*n* ≈ 240 total larvae) were well-powered to detect considerably smaller effects, including >90% power for a correlation of 0.21 (equal to 4.4% variance in sleep behavior explained by drug concentration [*R*^2^ = 0.044]).

## 3 Results

### 3.1 Summary

Visual inspection of plots of sleep/wake phenotypes across Ivabradine, Zatebradine hydrochloride and ZD7288 doses in some experiments suggested characteristics consistent with increased sleep on the day of drug administration. However, any differences observed with these drug compounds were smaller than the effect of melatonin. Melatonin increased sleep immediately after drug administration (see Supplementary Figures S2–S4). Each drug dose was tested on 33–36 zebrafish larvae in three biological replicates. Results of analyses performed as described in Section 2.4 for each drug are presented in more detail below.

### 3.2 Statistical analysis of Ivabradine screening

#### 3.2.1 Primary analysis of Ivabradine screening

Primary analyses of phenotypes as calculated within the time window between 30 min after Ivabradine administration (drug administration was performed at 5:00 pm) and 11 pm (beginning of the lights off period) are presented in Table 1. In ANOVA comparisons among groups, there was a difference in sleep latency (*p* = 0.020), with a shorter latency in the 0.1 μM group compared to DMSO (SMD = −0.321, *p* = 0.048). No differences in latency were observed between DMSO and other dosage groups, and results of linear and continuous dosage models were non-significant (see Table 1). Near significant differences—following ANOVA—were observed in average activity (*p* = 0.094), and average waking activity (*p* = 0.073). For both endpoints, continuous dosage models suggested some decreased activity for each 1 μM increase in Ivabradine, likely driven by the lower mean value in the 30 μM group. For comparison to differences between DMSO and Ivabradine doses, results of analyses comparing DMSO to the positive control melatonin during the same time period are shown in Supplementary Table S2. Strong differences between DMSO and melatonin were observed for all phenotypes (all *p* ≤ 0.006), with absolute standardized mean differences (SMDs) ranging from 0.49 for bout length to −1.15 for average waking activity.

**Table 1:** Sleep and activity of zebrafish larvae immediately after administration of Ivabradine doses and DMSO.

Phenotype	Adjusted Mean (95% CI)^†^							ANOVA *p*^‡^	Linear Model^§^		Dosage Model^¶^	
	DMSO	0.1 μM	0.3 μM	1.0 μM	4.5 μM	10 μM	30 μM		*β* (95% CI)	*p*	*β* (95% CI)	*p*
Total sleep, minutes	30.2 (21.8, 38.6)	33.7 (25.3, 42.1)	29.6 (21.3, 37.8)	22.2 (13.7, 30.8)	26.0 (17.6, 34.4)	30.2 (21.9, 38.6)	34.3 (26.0, 42.6)	0.447	0.08 (−1.50, 1.66)	0.924	0.18 (−0.13, 0.49)	0.253
Sleep bouts, number	13.1 (10.2, 16.1)	14.2 (11.2, 17.1)	12.8 (9.9, 15.7)	11.6 (8.6, 14.6)	10.3 (7.3, 13.2)	13.3 (10.4, 16.2)	15.8 (12.9, 18.7)	0.217	0.13 (−0.43, 0.69)	0.649	0.10 (−0.01, 0.21)	0.073
Sleep latency, minutes	127.8 (101.2, 154.4)	89.9 (63.6, 116.3)*(SMD = −0.321, p = 0.048)	150.8 (124.8, 176.9)	148.6 (121.6, 175.6)	114.8 (88.4, 141.2)	123.6 (97.2, 149.9)	111.4 (85.3, 137.5)	0.020	−0.67 (−5.70, 4.36)	0.793	−0.53 (−1.52, 0.458)	0.293
Bout length, minutes	2.03 (1.74, 2.31)	2.03 (1.76, 2.30)	2.20 (1.91, 2.49)	1.83 (1.52, 2.14)	1.99 (1.71, 2.27)	1.93 (1.64, 2.21)	2.02 (1.75, 2.30)	0.775	−0.015 (−0.068, 0.038)	0.578	−0.001 (−0.011, 0.010)	0.901
Avg. activity, sec/min	3.26 (3.05, 3.47)	3.27 (3.06, 3.48)	3.40 (3.19, 3.60)	3.33 (3.12, 3.54)	3.37 (3.16, 3.58)	3.22 (3.01, 3.43)	2.96 (2.75, 3.17)	0.094	−0.036 (−0.077, 0.006)	0.094	−0.012 (−0.020, −0.004)	0.003
Avg. wake act., sec/min	3.32 (3.12, 3.53)	3.37 (3.16, 3.58)	3.48 (3.28, 3.68)	3.38 (3.17, 3.59)	3.45 (3.25, 3.65)	3.29 (3.08, 3.49)	3.04 (2.83, 3.24)	0.073	−0.037 (−0.077, 0.004)	0.075	−0.012 (−0.020, −0.004)	0.002

Statistically significant associations (*p* < 0.05) shown in bold; ^†^Model estimated mean and 95% confidence interval, adjusted for replicate, experimental box, and baseline values of phenotype; ^‡^*p*-value from ANOVA testing whether there are any differences in phenotype among dosage groups; ^§^Results from linear model treating dose as an ordinal variable – *β* represents the expected change in phenotype associated with increasing to the next highest dosage group; ^¶^Results from continuous dosage model – *β* represents the expected change in phenotype for 1 μM increase in dose; *p < 0.05 compared to DMSO in pairwise comparisons (performed only when ANOVA *p* < 0.05). SMD and pairwise comparison results are indicated only for the sleep traits that displayed ANOVA *p* < 0.05.

#### 3.2.2 Secondary analysis of Ivabradine screening

Secondary analysis was performed for sleep phenotypes during the lights off period following one time Ivabradine administration in daytime (drug administration was performed at 5:00 pm). No significant differences among Ivabradine doses were observed based on ANOVA (Supplementary Table S3). A small increase in total sleep was observed in the continuous dosage model, with an increase of 0.71 min (95% CI: 0.09, 1.34) sleep for each 1 μM increase in Ivabradine (*p* = 0.025) (Supplementary Table S3). Results comparing DMSO and melatonin are again presented as a positive control (Supplementary Table S4). Small to moderate differences were observed with Melatonin in the number (SMD = −0.49, *p* = 0.002) and length (SMD = 0.38, *p* = 0.007) of sleep bouts, but there were no differences in total sleep or sleep latency.

### 3.3 Statistical analysis of Zatebradine hydrochloride screening

#### 3.3.1 Primary analysis of Zatebradine hydrochloride screening

Comparisons of sleep and activity patterns among drug doses immediately after Zatebradine Hydochloride administration are presented in Table 2. Differences were observed among groups for average activity (*p* = 0.024) and average waking activity (*p* = 0.030), but there were no differences in other phenotypes based on ANOVA. Compared to DMSO, the 30 μM dose group showed significantly lower average activity (SMD = −0.43, *p* = 0.032) and average waking (SMD = −0.40, *p* = 0.041) activity. These differences are reflected in significant associations in continuous dose models for each phenotype, but only trending results in linear models (Table 2). An association (*p* = 0.034) in the dosage model was also observed for sleep latency, with each 1 μM increase in Zatebradine Hydochloride associated with a 1.61 min decrease (95% CI: −3.09, −0.13). We again observed significant differences in all phenotypes when comparing DMSO to melatonin as a positive control (Supplementary Table S5), with absolute SMDs ranging from 0.53 for sleep bout length to −1.42 for average activity and average waking activity.

**Table 2:** Sleep and activity of zebrafish larvae immediately after administration of Zatebradine hydrochloride doses and DMSO.

Phenotype	Adjusted Mean (95% CI)^†^							ANOVA *p*^‡^	Linear model^§^		Dosage model^¶^	
	DMSO	0.1 μM	0.3 μM	1.0 μM	4.5 μM	10 μM	30 μM		*β* (95% CI)	*p*	β (95% CI)	*p*
Total sleep, minutes	22.8 (11.8, 33.8)	25.2 (14.2, 36.2)	20.5 (9.5, 31.4)	29.1 (18.2, 40.0)	25.0 (14.2, 35.8)	22.3 (11.3, 33.3)	33.3 (22.3, 44.3)	0.714	1.08 (−0.99, 3.14)	0.305	0.28 (−0.126, 0.686)	0.176
Sleep bouts, number	11.0 (6.6, 15.5)	13.2 (8.7, 17.7)	10.5 (6.0, 15.0)	14.4 (10.0, 18.8)	12.7 (8.3, 17.1)	13.1 (8.6, 17.5)	15.4 (10.8, 19.9)	0.757	0.53 (−0.32, 1.37)	0.220	0.10 (−0.064, 0.273)	0.225
Sleep latency, minutes	147.0 (107.1, 186.8)	137.5 (97.7, 177.4)	169.7 (129.9, 209.6)	164.2 (125.0, 203.4)	180.1 (140.9, 219.4)	159.1 (119.2, 198.9)	106.4 (66.5, 146.2)	0.183	−2.41 (−9.99, 5.16)	0.531	−1.61 (−3.087, −0.126)	0.034
Bout length, minutes	1.85 (1.45, 2.26)	2.07 (1.68, 2.46)	1.97 (1.57, 2.38)	1.86 (1.43, 2.29)	1.75 (1.37, 2.12)	2.14 (1.73, 2.54)	1.62 (1.22, 2.02)	0.560	−0.030 (−0.106, 0.046)	0.434	−0.009 (−0.024, 0.006)	0.228
Avg. activity, sec/min	3.15 (2.81, 3.48)	3.22 (2.89, 3.56)	3.44 (3.11, 3.78)	3.40 (3.07, 3.73)	3.31 (2.98, 3.64)	3.21 (2.87, 3.54)	2.62 (2.28, 2.96)*(SMD = −0.43, *p* = 0.032)	0.024	−0.059 (−0.123, 0.005)	0.072	−0.022 (−0.035, −0.010)	0.001
Avg. wake act., sec/min	3.20 (2.87, 3.52)	3.28 (2.95, 3.61)	3.49 (3.16, 3.82)	3.46 (3.14, 3.79)	3.36 (3.04, 3.69)	3.24 (2.91, 3.57)	2.71 (2.37, 3.04)*(SMD = −0.40, p = 0.041)	0.030	−0.057 (−0.119, 0.006)	0.078	−0.021 (−0.034, −0.009)	0.001

Statistically significant associations (*p* < 0.05) shown in bold; ^†^Model estimated mean and 95% confidence interval, adjusted for replicate and baseline values of phenotype; ^‡^*p*-value from ANOVA testing whether there are any differences in phenotype among dosage groups; ^§^Results from linear model treating dose as an ordinal variable – *β* represents the expected change in phenotype associated with increasing to the next highest dosage group; ^¶^Results from continuous dosage model – *β* represents the expected change in phenotype for 1 μM increase in dose. *p < 0.05 compared to DMSO in pairwise comparisons (performed only when ANOVA *p* < 0.05). SMD and pairwise comparison results are indicated only for the traits that displayed ANOVA *p* < 0.05.

#### 3.3.2 Secondary analysis of Zatebradine hydrochloride screening

Secondary analyses were performed for sleep phenotypes in the lights off period following one time Zatebradine Hydochloride administration in daytime (drug administration was performed at 5:00 pm) (Supplementary Table S6). There were no significance among group differences based on ANOVA. There was statistically significant (*p* = 0.025) evidence of a small increase in the number of sleep bouts for a 1 μM increase in dosage. There were no differences between DMSO and Melatonin in the lights off period for these phenotypes in this experiment (Supplementary Table S7).

### 3.4 Statistical analysis of ZD7288 screening

#### 3.4.1 Primary analysis of ZD7288 screening

Comparisons of sleep and activity patterns across doses immediately after ZD7288 administration are presented in Table **3**. No differences were observed based on ANOVA results. In dose response analyses of bout length, both the linear model and dosage model indicated longer bouts with increased dose of ZD7288 (*p* = 0.021 and *p* = 0.005). The linear model showed an increased bout length of 0.13 min per increase in dosage group (*p* = 0.021) and the dosage model showed an increased bout length of 0.03 min per 1 μM increase (*p* = 0.005). As in previous experiments, comparisons between DMSO and melatonin as a positive control demonstrated significant differences across all phenotypes (Supplementary Table S8), with absolute SMDs ranging from 0.82 for sleep bout length to 1.52 for the number of sleep bouts.

**Table 3:** Sleep and activity of zebrafish larvae immediately after administration of ZD7288 doses and DMSO.

Phenotype	Adjusted Mean (95% CI)^†^							ANOVA *p*^‡^	Linear model^§^		Dosage model^¶^	
	DMSO	0.1 μM	0.3 μM	1.0 μM	4.5 μM	10 μM	30 μM		*β* (95% CI)	*p*	*β* (95% CI)	*p*
Total sleep, minutes	25.3 (12.7, 38.0)	25.1 (12.2, 38.0)	28.4 (15.7, 41.0)	17.7 (4.8, 30.5)	43.9 (31.1, 56.8)	24.5 (11.7, 37.3)	33.9 (20.4, 47.3)	0.135	1.41 (−1.09, 3.90)	0.268	0.26 (−0.25, 0.76)	0.320
Sleep bouts, number	12.0 (7.6, 16.5)	10.3 (5.8, 14.8)	13.1 (8.7, 17.5)	11.4 (6.90, 15.95)	20.0 (15.5, 24.5)	11.3 (6.8, 15.7)	11.2 (6.4, 16.0)	0.052	0.24 (−0.65, 1.14)	0.592	−0.05 (−0.24, 0.13)	0.585
Sleep latency, minutes	95.1 (60.8, 129.4)	132.5 (97.7, 167.3)	106.9 (72.6, 141.2)	133.3 (98.3, 168.2)	94.1 (59.0, 129.3)	125.1 (90.0, 160.1)	84.8 (49.8, 119.9)	0.286	−1.91 (−8.50, 4.68)	0.569	−0.89 (−2.19, 0.42)	0.181
Bout length, minutes	1.60 (1.01, 2.18)	2.09 (1.46, 2.72)	1.70 (1.12, 2.29)	1.67 (1.03, 2.30)	2.02 (1.47, 2.58)	1.93 (1.28, 2.58)	2.64 (2.12, 3.16)	0.132	0.128 (0.020, 0.236)	0.021	0.029 (0.009, 0.049)	0.005
Avg. activity, sec/min	3.51 (3.18, 3.85)	3.63 (3.29, 3.96)	3.23 (2.91, 3.56)	3.20 (2.88, 3.53)	3.02 (2.69, 3.35)	3.36 (3.04, 3.69)	3.02 (2.68, 3.37)	0.138	−0.074 (−0.144, −0.004)	0.039	−0.009 (−0.023, 0.004)	0.170
Avg. wake act., sec/min	3.58 (3.27, 3.90)	3.70 (3.38, 4.03)	3.31 (3.00, 3.62)	3.25 (2.94, 3.56)	3.14 (2.82, 3.46)	3.40 (3.09, 3.72)	3.13 (2.80, 3.46)	0.167	−0.071 (−0.138, −0.003)	0.040	−0.008 (−0.021, 0.005)	0.214

Statistically significant associations (*p* < 0.05) shown in bold; ^†^Model estimated mean and 95% confidence interval, adjusted for replicate and baseline values of phenotype; ^‡^*p*-value from ANOVA testing whether there are any differences in phenotype among dosage groups; ^§^Results from linear model treating dose as an ordinal variable – *β* represents the expected change in phenotype associated with increasing to the next highest dosage group; ^¶^Results from continuous dosage model – *β* represents the expected change in phenotype for 1 μM increase in dose.

#### 3.4.2 Secondary analysis of ZD7288 screening

Secondary analyses were performed for sleep phenotypes in the lights off period following one time ZD7288 administration in daytime (drug administration was performed at 5:00 pm) (Supplementary Table S9). In ANOVA comparisons, differences among dosage groups were observed for total sleep (*p* = 0.036), number of sleep bouts (*p* = 0.0003) and sleep bout length (*p* = 0.003); there was a near significant difference in sleep latency (*p* = 0.064). Interestingly, differences among groups were driven by an increase in total sleep (SMD = 0.53, *p* = 0.008), decreased number of sleep bouts (SMD = −0.75, *p* = 0.001), and increased sleep bout length (SMD = 0.54, *p* = 0.003) within the 4.5 μM group compared to DMSO. In addition, the 10 μM group demonstrated fewer sleep bouts than DMSO (SMD = −0.49, *p* = 0.022). These associations between sleep phenotypes and moderate doses of ZD7288 are reflected in significant associations in the linear dose response analyses (Supplementary Table S9). There were no differences between DMSO and melatonin for these phenotypes in the lights off period (Supplementary Table S10).

## 4 Discussion

Screening effects of HCN channel blockers on sleep/wake behavior of zebrafish larvae resulted in shorter latency to sleep at 0.1 μM dose of Ivabradine, moderate reductions in average activity at 30 μM dose of Zatebradine Hydrochloride, and more consolidated sleep at 4.5 μM dose of ZD7288 as a result of ANOVA analysis in our study. Among these results, reduction in activity following Zatebradine Hydrochoride administration was supported by dosage model and increased sleep following ZD7288 administration was supported by linear model. Since significant associations in ANOVA were not supported by both dose–response analyses (ie. linear model and dosage model), we conclude that a single dose of each of the tested drugs may be driving the overall results, rather than exerting a robust effect on sleep/wake behavior. Interestingly, more consolidated sleep was detected at a single middle dose of ZD7288 (i.e. 4.5 μM) at nighttime sleep. Indeed, sleep amount in this dose group was the highest among others and bout length was higher at this dose compared to the DMSO group in daytime sleep, whereas these differences did not reach significance in daytime sleep and reached significance at nighttime. This might be an optimum dose of this compound to affect sleep in zebrafish.

Our findings associated with sleep and activity parameters were in the same direction; shorter latency to sleep indicates falling asleep faster, reduced activity and increased sleep imply increased amount of sleep however each compound impacted a different parameter of sleep or activity. There were different reports on effects of antagonists of HCN channels such as decreased (Huang et al., 2020) and increased wakefulness (Li et al., 2010) in mouse models, fragmented sleep (Gonzalo-Gomez et al., 2012) and no change in total sleep duration (Gonzalo-Gomez et al., 2012) in a *Drosophila* model. We observed highly diverse effects of three different HCN channel blocking agents. Since antagonists of HCN channels are utilized to lower heart rate, administration of these compounds on zebrafish larva may affect their locomotor behavior, cardiovascular system and other peripheral systems in a non-specific manner. In addition, zebrafish behavior demonstrates high variability. We normalized the behavior data against the data of the same zebrafish larva at the same time of day from the previous day and we compared sleep/wake behavior of dose groups of animals with DMSO exposed group following that normalization. This approach was designed to overcome inter-individual variability. Moreover, melatonin, which was administered at an equal dose to the lowest dose of the tested drugs, robustly and rapidly increased sleep. Therefore, we conclude that although certain sleep and activity parameters were affected by administration of particular doses of the tested compounds, their effects on sleep/wake behavior in zebrafish were not as robust as that of melatonin.

The half-life of the three HCN channel blocker compounds range between two-three hours (Valenzuela et al., 1996; Chaplan et al., 2003; Tse and Mazzola, 2015). Compounds were administered at 5 pm and our primary analysis took place between 05:30–11 pm. Thus, our primary analysis time window included half-life of the tested three HCN channel blockers. Ivabradine does not cross the blood brain barrier (Savelieva and Camm, 2008). Zatebradine hydrochloride passes blood brain barrier (Kruger et al., **2000**). Ability of ZD7288 to pass blood brain barrier is not known (Zhong and Darmani, 2021). Blood brain barrier is sealed by day 5 into development in zebrafish (O’brown et al., 2019). We administered HCN channel blockers to zebrafish larvae at 6 dpf (days post fertilization). Therefore, we mimicked the conditions of how humans take HCN channel blockers in our study.

Zatebradine hydrochloride inhibits inward current in Purkinje cells (Valenzuela et al., 1996). In wild type mice, cerebellar activity was lower in NREM sleep compared to that in wakefulness and it was reported to be elevated during REM sleep (Zhang et al., 2020). Also, Purkinje cells were active before transitioning from sleep to wakefulness (Zhang et al., 2020). Reduction in activity following Zatebradine hydrochloride administration in the current study may point out decreased wakefulness and this finding is in line with the aforementioned reports, most likely through a mechanism affecting Purkinje cells. Additionally, ZD7288 was shown to suppress glutamate release from the hippocampus in rats (Zhang et al., 2016). Given that chemogenetic inhibition of glutamate, which is an excitatory neurotransmitter, increased NREM sleep and decreased wakefulness in mice (Kroeger et al., 2017) and more consolidated sleep in zebrafish was observed as a response to a single middle dose of ZD7288 in our study, we suggest that this effect might have been obtained due to inhibition of glutamate release. Although Ivabradine does not cross the blood brain barrier, it inhibits inward current in peripheral and autonomic somatosensory neurons (Scridon et al., 2021), thereby this effect might explain shorter latency to daytime sleep immediately after drug administration. Moreover, HCN channel blocker compounds including Ivabradine (Demontis et al., 2009), Zatebradine hydrochloride (Satoh and Yamada, 2002) and ZD7288 (Satoh and Yamada, 2000) inhibit Ih in HCN channels especially HCN1 in rod photoreceptors which contributes to photoreceptor degeneration (Schon et al., 2016). Given that zebrafish are highly responsive to light in sleep regulation (Jones, 2007), blockade of photoreceptors in retina might have a role in the phenotypes we observed in our study.

Use of zebrafish as a model organism provided us with the opportunity to assess effects of compounds on the whole brain instead of focusing on one brain region at a time (Li et al., 2010; Huang et al., 2020). Zebrafish is a diurnal organism like humans. This was another advantage of using zebrafish over using a mouse model as mice are nocturnal. *Drosophila* is an invertebrate model (Gonzalo-Gomez et al., 2012). Since zebrafish is a vertebrate model, it possesses more evolutionarily conserved features with mammals compared to *Drosophila* such as nervous system and neuropharmacology (Oikonomou and Prober, 2017). Zebrafish is an attractive *in vivo* model to perform drug repurposing studies (Cousin et al., 2014; Wittmann et al., 2015; Sourbron et al., 2019). In this study, we tested effects of HCN channel blocker compounds, which are used as pharmacological tools to reduce heart rate, on sleep/wake behaviors in zebrafish larvae. Blocking HCN channels has been suggested to be effective in pain treatment (Ramirez et al., 2018). Zebrafish drug screening libraries (Rihel et al., 2010a) can be utilized to identify the pathways through which HCN channel blocker compounds exert their functions associated with alleviating neuropathic pain.

Waking activity data is utilized to assess health status of the zebrafish larvae in sleep/wake assays (Rihel et al., 2010a). We did not see large changes in waking activity following the administration of HCN channel blockers. Average waking activity across Ivabradine and ZD7288 doses were not significantly different from DMSO controls immediately after drug administration. Zatebradine hydrochloride screening demonstrated differences among groups in average activity and average waking activity. Small to moderate effects were observed in both average waking activity (SMD = −0.40, *p* = 0.041) and average activity (SMD = −0.43, *p* = 0.032) in the 30 μM dose group of Zatebradine hydrochloride compared to DMSO, indicating that differences in average waking activity is in line with that in average activity. Confidence intervals of both parameters mostly overlap. Therefore, we conclude that the doses of three compounds administered were not toxic and zebrafish larvae were healthy during the assessed period of time.

The limitations of our model of choice might be due to the method of drug administration. Drug compounds dissolved in DMSO were pipetted into individual wells of a 96 well plate in which individual larva swims rather than directly administering it such as injecting. However, this is the standard method of drug screening assays in zebrafish (Rihel et al., 2010a; Mosser et al., 2019).

Our study is the first report of testing effects of HCN channel blockers in zebrafish to our knowledge. We also displayed and analyzed effects of melatonin in zebrafish larvae as a positive control. Ivabradine, Zatebradine hydrochloride and ZD7288 do not work selectively on HCN channel subunits (Novella Romanelli et al., 2016). CRISPR/Cas9 screening of the genes at the founder generation is a favorable approach since it allows researchers to identify behavioral phenotypes rapidly on a gene knockout (Kroll et al., 2021; Zimmerman et al., 2022). Each HCN channel subunit might be targeted genetically using CRISPR/Cas9 screening technique in future studies to dissect the role of each gene in sleep/wake behavior.

## Data availability statement

The raw data supporting the conclusions of this article will be made available by the authors, without undue reservation.

## Ethics statement

The animal study was approved by the University of Pennsylvania Institutional Animal Care and Use Committee. The study was conducted in accordance with the local legislation and institutional requirements.

## Author contributions

FD-B: Conceptualization, Data curation, Formal analysis, Investigation, Methodology, Writing – original draft, Writing – review & editing. SPS: Conceptualization, Funding acquisition, Writing – original draft, Writing – review & editing. BTK: Formal analysis, Writing – original draft, Writing – review & editing. AJZ: Writing – original draft, Writing – review & editing. OJV: Writing – original draft, Writing – review & editing. CMP: Writing – original draft, Writing – review & editing. GB: Writing – original draft, Writing – review & editing. MHP: Funding acquisition, Writing – original draft, Writing – review & editing.
