# Supplementary material for: Screening effects of HCN channel blockers on sleep/wake behavior in zebrafish
Source: Front Neurosci. 2024 Mar 19;18:1375484. doi: 10.3389/fnins.2024.1375484 (PMC10986788; doi:10.3389/fnins.2024.1375484)
Supplement: Supplementary file 1 [file Data_Sheet_1.PDF]

Supplementary Figures and Tables

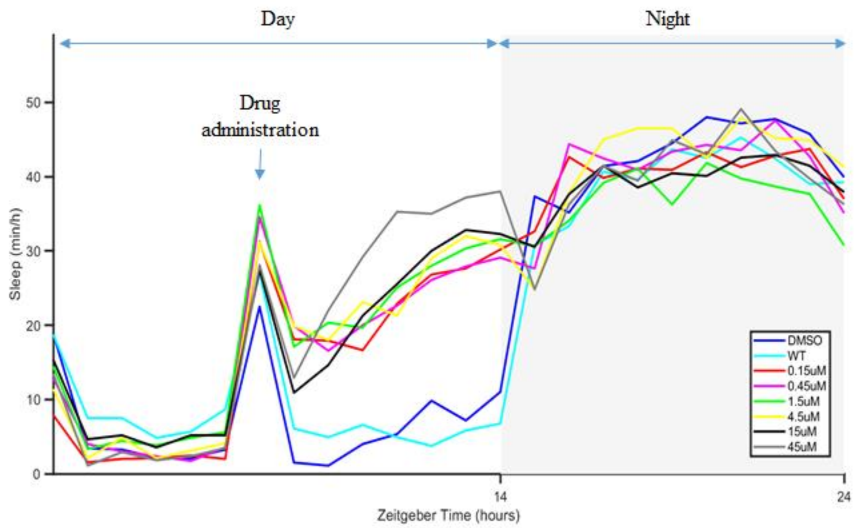

Figure S1. Dose response curve performed to test effects of six different doses of melatonin ranging between 0.15-45 micromolar on sleep/wake behavior of zebrafish larva compared to DMSO and wild type controls. Large peaks occur at the time of melatonin administration when the lid of the equipment is lifted and closed back on. Melatonin rapidly increases sleep at all of the tested doses. n:12 per group.

Table S1. Definitions of Key Behavioral Phenotypes in Drug Screening Study

Phenotypes	Definition
**Total Sleep**	Total sleep in minutes. Sleep is defined as any one-minute period of inactivity with less than 0.5 second of total movement of zebrafish larva.
**Sleep Bouts**	A continuous sequence of sleep minutes. Total sleep bouts is the bout numbers in a given period.
**Sleep Latency**	Length of time (in minutes) spent until the first sleep bout starts.
**Bout Length**	Average duration of a sleep bout (in minutes).
**Average Activity**	Average activity (seconds/minute) calculated by dividing total activity by total recording minutes
**Average Waking Activity**	Average waking activity (seconds/minute) calculated by dividing total activity by the total waking minutes

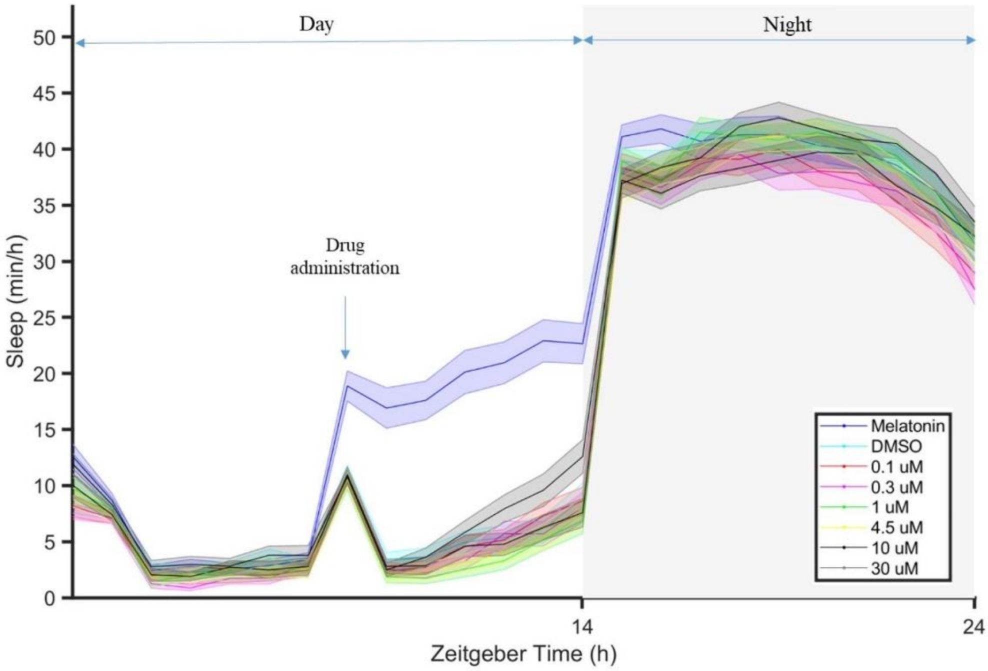

Figure S2. Effect of Ivabradine (Corlanor) on sleep of zebrafish larvae. Different final concentrations of this drug compound ranging between 0.1 μM and 30 μM, DMSO control and 0.1 μM melatonin were added to the wells containing 6 days post fertilization larvae. Trending but non-significant increase in sleep duration, likely driven by the higher mean value in the 30 μM Ivabradine group can be observed. Total n:550, melatonin n:67, DMSO n:68, 0.1 μM n:69, 0.3 μM n:71, 1 μM n:66, 4.5 μM n:69, 10 μM n:69, 30 μM n:71, n: number of animals. Graphs are presented as mean ± SEM.

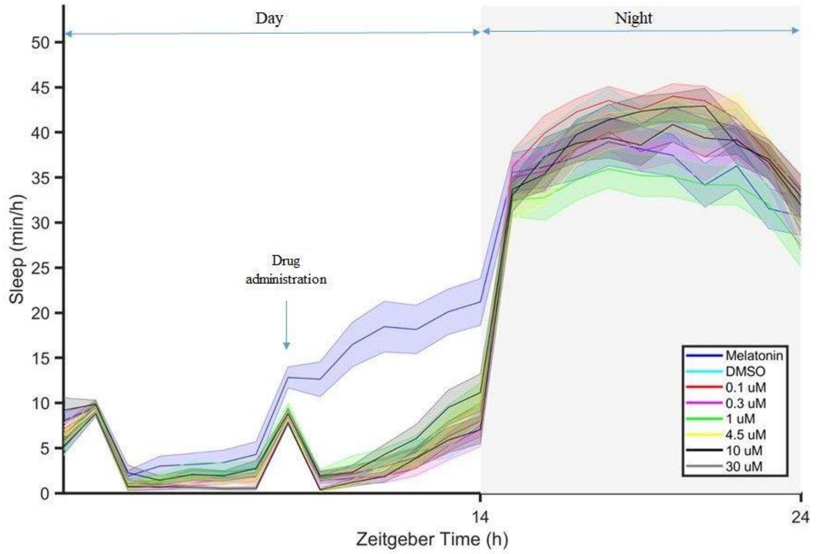

Figure S3. Effect of Zatebradine hydrochloride on sleep of zebrafish larvae. Different final concentrations of this drug compound ranging between 0.1 µM and 30 µM, DMSO control and 0.1 µM melatonin were added to wells containing 6 days post fertilization larvae. The 30 µM dose group showed an increasing trend in sleep duration however overall ANOVA was not significant. Total n:275, melatonin n:35, DMSO n:34, 0.1 µM n:34, 0.3 µM n:34, 1 µM n:35, 4.5 µM n:35, 10 µM n:34, 30 µM n:34, n: number of animals. Graphs are presented as mean ± SEM.

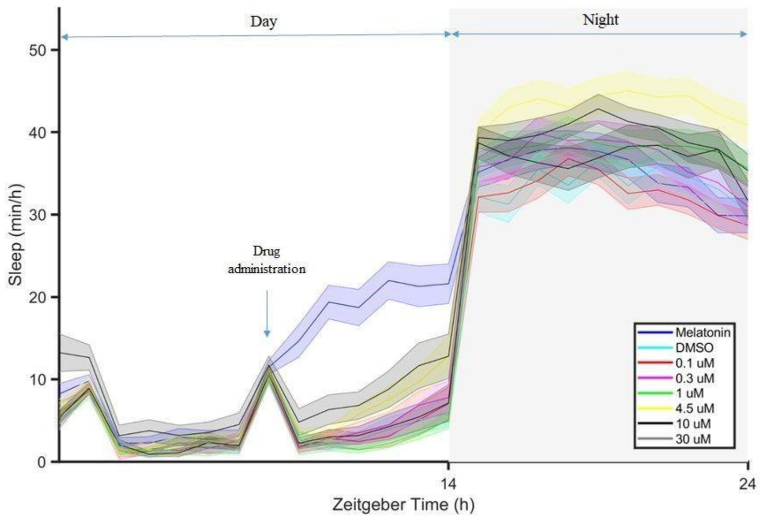

Figure S4. Effect of ZD7288 on sleep of zebrafish larvae. Different final concentrations of this drug compound ranging between 0.1 µM and 30 µM, DMSO control and 0.1 µM melatonin were added to wells containing 6 days post fertilization larvae. The 4.5 and 30 µM doses of ZD7288 demonstrated increased sleep amount immediately after drug was administered but overall ANOVA was not significant. Total sleep was significantly increased at 4.5 µM dose compared to DMSO during lights off period. Total n:275, melatonin n:35, DMSO n:35, 0.1 µM n:34, 0.3 µM n:35, 1 µM n:34, 4.5 µM n:34, 10 µM n:34, 30 µM n:34, n:number of animals. Graphs are presented as mean ± SEM.

Table S2. Sleep and Activity of Zebrafish Larvae Immediately After Melatonin Administration as a Positive Control in Ivabradine Screening Assay

Phenotype	Adjusted Mean (95% CI)†		Difference (95% CI)‡	p§
	DMSO	Melatonin		
**Total Sleep, minutes**	35.6 (22.1, 49.1)	116.5 (102.9, 130.1) (SMD:1.01)	**80.9 (61.7, 100.1)**	**<0.0001**
**Sleep Bouts, number**	14.7 (10.4, 19.0)	41.4 (37.1, 45.8) (SMD:1.11)	**26.7 (20.5, 32.8)**	**<0.0001**
**Sleep Latency, minutes**	121.0 (99.5, 142.5)	21.7 (0.0, 43.4) (SMD:-0.094)	**-99.3 (-129.9, -68.7)**	**<0.0001**
**Bout Length, minutes**	2.04 (1.69, 2.39)	2.73 (2.40, 3.06) (SMD:0.49)	**0.69 (0.21, 1.17)**	**0.006**
**Avg. Activity, sec/min**	3.22 (3.01, 3.42)	1.28 (1.07, 1.48) (SMD:-1.14)	**-1.94 (-2.24, -1.64)**	**<0.0001**
**Avg. Wake Act., sec/min**	3.30 (3.10, 3.51)	1.35 (1.14, 1.56) (SMD:-1.15)	**-1.95 (-2.25, -1.65)**	**<0.0001**

Statistically significant differences (p<0.05) shown in **bold**; †Model estimated mean and 95% confidence interval, adjusted for replicate, experimental box, and baseline values of phenotype; ‡Adjusted mean difference and 95% CI between melatonin and DMSO groups; §p-value comparing phenotype between DMSO and Melatonin groups.

Table S3. Sleep of Zebrafish Larvae During the Lights Off Period Following the Daytime Administration of Ivabradine Doses and DMSO

Phenotype	Adjusted Mean (95% CI)†							ANOVA p‡	Linear Model§		Dosage Model¶	
	DMSO	0.1 µM	0.3 µM	1.0 µM	4.5 µM	10 µM	30 µM		β (95% CI)	p	β (95% CI)	p
**Total Sleep, minutes**	377.5 (360.4, 394.6)	375.2 (358.2, 392.2)	366.6 (349.9, 383.3)	384.4 (367.1, 401.8)	377.5 (360.6, 394.5)	366.5 (349.5, 383.5)	398.9 (382.1, 415.6)	0.118	2.12 (-1.08, 5.33)	0.194	**0.71 (0.09, 1.34)**	**0.025**
**Sleep Bouts, number**	80.6 (76.9, 84.3)	74.5 (70.8, 78.2)	74.4 (70.7, 78.0)	78.4 (74.6, 82.1)	78.2 (74.6, 81.9)	78.8 (75.1, 82.5)	80.7 (77.1, 84.4)	0.071	0.47 (-0.23, 1.17)	0.185	0.13 (-0.007, 0.267)	0.063
**Sleep Latency, minutes**	6.86 (6.05, 7.68)	5.77 (4.95, 6.58)	6.26 (5.46, 7.07)	5.67 (4.84, 6.50)	6.84 (6.02, 7.65)	6.90 (6.09, 7.72)	6.41 (5.61, 7.22)	0.160	0.054 (-0.100, 0.208)	0.489	0.010 (-0.021, 0.040)	0.532
**Bout Length, minutes**	5.02 (4.56, 5.48)	5.29 (4.84, 5.75)	5.32 (4.87, 5.77)	5.15 (4.69, 5.62)	5.20 (4.75, 5.66)	5.05 (4.60, 5.51)	5.58 (5.13, 6.03)	0.678	0.038 (-0.047, 0.124)	0.380	0.012 (-0.005, 0.028)	0.176

Statistically significant associations ($p<0.05$) shown in **bold**; †Model estimated mean and 95% confidence interval, adjusted for replicate, experimental box, and baseline values of phenotype; ‡p-value from ANOVA testing whether there are any differences in phenotype among dosage groups; §Results from linear model treating dose as an ordinal variable – β represents the expected change in phenotype associated with increasing to the next highest dosage group; ¶Results from continuous dosage model – β represents the expected change in phenotype for 1 µM increase in dose;*$p<0.05$ compared to DMSO in pairwise comparisons (performed only when ANOVA $p<0.05$).

Table S4. Sleep of Zebrafish Larvae During the Lights Off Period Following the Daytime Melatonin Administration as a Positive Control in Ivabradine Screening Assay

**Phenotype**	**Adjusted Mean (95% CI)†**		**Difference (95% CI)‡**	**p§**
	**DMSO**	**Melatonin**		
**Total Sleep, minutes**	379.7 (362.9, 396.5)	394.7 (377.7, 411.6)	15.0 (-8.9, 38.8)	0.218
**Sleep Bouts, number**	81.0 (77.2, 84.8)	72.5 (68.7, 76.3) (SMD = -0.49)	**-8.5 (-13.9, -3.1)**	**0.002**
**Sleep Latency, minutes**	6.75 (5.95, 7.54)	6.50 (5.69, 7.30)	-0.25 (-1.38, 0.89)	0.667
**Bout Length, minutes**	5.01 (4.53, 5.49)	5.96 (5.47, 6.44) (SMD = 0.38)	**0.95 (0.27, 1.63)**	**0.007**

Statistically significant differences ($p<0.05$) shown in **bold**; †Model estimated mean and 95% confidence interval, adjusted for replicate, experimental box, and baseline values of phenotype; ‡Adjusted mean difference and 95% CI between melatonin and DMSO groups; §p-value comparing phenotype between DMSO and Melatonin groups. SMD values are indicated only for the sleep traits that displayed ANOVA $p<0.05$.

Table S5. Sleep and Activity of Zebrafish Larvae Immediately After Melatonin Administration as a Positive Control in Zatebradine Hydrochloride Screening Assay

**Phenotype**	**Adjusted Mean (95% CI)†**		**Difference (95% CI)‡**	**p§**
	**DMSO**	**Melatonin**		
**Total Sleep, minutes**	20.9 (1.4, 40.3)	108.9 (89.4, 128.3) (SMD:1.19)	**88.0 (60.3, 115.6)**	**<0.0001**
**Sleep Bouts, number**	11.5 (4.8, 18.2)	42.5 (35.8, 49.2) (SMD:1.23)	**31.0 (21.55, 40.52)**	**<0.0001**
**Sleep Latency, minutes**	144.1 (110.8, 177.3)	31.7 (-1.5, 65.0) (SMD:-1)	**-112.4 (-159.5, -65.3)**	**<0.0001**
**Bout Length, minutes**	1.82 (1.49, 2.15)	2.31 (2.01, 2.61) (SMD:0.53)	**0.49 (0.05, 0.94)**	**0.031**
**Avg. Activity, sec/min**	3.12 (2.85, 3.38)	1.15 (0.89, 1.41) (SMD:-1.42)	**-1.97 (-2.34, -1.59)**	**<0.0001**
**Avg. Wake Act., sec/min**	3.17 (2.91, 3.43)	1.22 (0.96, 1.48) (SMD:-1.42)	**-1.95 (-2.32, -1.58)**	**<0.0001**

Statistically significant differences (p<0.05) shown in **bold**; †Model estimated mean and 95% confidence interval, adjusted for replicate and baseline values of phenotype; ‡Adjusted mean difference and 95% CI between melatonin and DMSO groups; §p-value comparing phenotype between DMSO and Melatonin groups.

Table S6. Sleep of Zebrafish Larvae During the Lights Off Period Following the Daytime Administration of Zatebradine Hydrochloride Doses and DMSO

Phenotype	Adjusted Mean (95% CI)†							ANOVA p‡	Linear Model§		Dosage Model¶	
	DMSO	0.1 µM	0.3 µM	1.0 µM	4.5 µM	10 µM	30 µM		β (95% CI)	p	β (95% CI)	p
**Total Sleep, minutes**	386.2 (359.3, 413.2)	393.7 (366.4, 421.0)	369.4 (342.5, 396.4)	337.1 (310.5, 363.7)	384.6 (357.9, 411.2)	377.5 (350.6, 404.4)	388.6 (361.7, 415.5)	0.068	-0.25 (-5.45, 4.95)	0.925	0.52 (-0.50, 1.53)	0.319
**Sleep Bouts, number**	78.2 (73.0, 83.3)	79.0 (73.8, 84.1)	76.0 (70.9, 81.1)	76.7 (71.7, 81.8)	77.1 (72.1, 82.2)	82.8 (77.7, 87.9)	83.3 (78.2, 88.5)	0.264	0.87 (-0.10, 1.83)	0.079	**0.22 (0.03, 0.41)**	**0.025**
**Sleep Latency, minutes**	7.45 (5.94, 8.97)	8.29 (6.78, 9.81)	7.87 (6.36, 9.38)	8.93 (7.43, 10.44)	7.72 (6.23, 9.22)	7.11 (5.59, 8.62)	7.02 (5.51, 8.53)	0.582	-0.139 (-0.424, 0.146)	0.338	-0.039 (-0.095, 0.016)	0.166
**Bout Length, minutes**	5.28 (4.63, 5.93)	5.35 (4.69, 6.01)	5.11 (4.47, 5.76)	4.90 (4.26, 5.54)	5.27 (4.63, 5.91)	4.74 (4.09, 5.39)	5.08 (4.43, 5.72)	0.841	-0.059 (-0.181, 0.063)	0.341	-0.005 (-0.029, 0.019)	0.667

Statistically significant associations (p<0.05) shown in **bold**; †Model estimated mean and 95% confidence interval, adjusted for replicate and baseline values of phenotype; ‡p-value from ANOVA testing whether there are any differences in phenotype among dosage groups; §Results from linear model treating dose as an ordinal variable – β represents the expected change in phenotype associated with increasing to the next highest dosage group; ¶Results from continuous dosage model – β represents the expected change in phenotype for 1 µM increase in dose;*p<0.05 compared to DMSO in pairwise comparisons (performed only when ANOVA p<0.05).

Table S7. Sleep of Zebrafish Larvae During the Lights Off Period Following the Daytime Melatonin Administration as a Positive Control in Zatebradine Hydrochloride Screening Assay

Phenotype	Adjusted Mean (95% CI)†		Difference (95% CI)‡	p§
	DMSO	Melatonin		
**Total Sleep, minutes**	382.6 (353.7, 411.5)	358.0 (329.2, 386.9)	-24.6 (-65.6, 16.5)	0.237
**Sleep Bouts, number**	79.2 (73.8, 84.7)	78.7 (73.2, 84.1)	-0.5 (-8.3, 7.2)	0.892
**Sleep Latency, minutes**	7.34 (5.44, 9.24)	8.55 (6.65, 10.45)	1.21 (-1.48, 3.90)	0.372
**Bout Length, minutes**	5.19 (4.65, 5.73)	4.59 (4.05, 5.13)	-0.60 (-1.37, 0.17)	0.123

†Model estimated mean and 95% confidence interval, adjusted for replicate and baseline values of phenotype; ‡Adjusted mean difference and 95% CI between melatonin and DMSO groups; §p-value comparing phenotype between DMSO and Melatonin groups.

Table S8. Sleep and Activity of Zebrafish Larvae Immediately After Melatonin Administration as a Positive Control in ZD7288 Screening Assay

Phenotype	Adjusted Mean (95% CI)†		Difference (95% CI)‡	p§
	DMSO	Melatonin		
**Total Sleep, minutes**	22.5 (4.6, 40.4)	110.3 (92.4, 128.2) (SMD:1.27)	**87.8 (62.2, 113.3)**	**<0.0001**
**Sleep Bouts, number**	10.6 (5.7, 15.5)	44.3 (39.4, 49.2) (SMD:1.52)	**33.7 (26.7, 40.7)**	**<0.0001**
**Sleep Latency, minutes**	94.7 (69.9, 119.5)	12.4 (-12.4, 37.2) (SMD:-0.97)	**-82.3 (-117.7, -46.9)**	**<0.0001**
**Bout Length, minutes**	1.60 (1.24, 1.95)	2.39 (2.07, 2.71) (SMD:0.82)	**0.80 (0.32, 1.28)**	**0.002**
**Avg. Activity, sec/min**	3.82 (3.50, 4.14)	1.26 (0.94, 1.57) (SMD:-1.44)	**-2.56 (-3.03, -2.10)**	**<0.0001**
**Avg. Wake Act., sec/min**	3.90 (3.59, 4.20)	1.37 (1.07, 1.68) (SMD:-1.45)	**-2.52 (-2.97, -2.08)**	**<0.0001**

Statistically significant differences (p<0.05) shown in **bold**; †Model estimated mean and 95% confidence interval, adjusted for replicate and baseline values of phenotype; ‡Adjusted mean difference and 95% CI between melatonin and DMSO groups; §p-value comparing phenotype between DMSO and Melatonin groups.

Table S9. Sleep of Zebrafish Larvae During the Lights Off Period Following the Daytime Administration of ZD7288 Doses and DMSO

Phenotype	Adjusted Mean (95% CI)†							ANOVA p‡	Linear Model§		Dosage Model¶	
	**DMSO**	**0.1 µM**	**0.3 µM**	**1.0 µM**	**4.5 µM**	**10 µM**	**30 µM**		**β (95% CI)**	**p**	**β (95% CI)**	**p**
**Total Sleep, minutes**	356.8 (328.6, 385.0)	340.1 (311.7, 368.5)	371.1 (343.3, 398.8)	374.8 (346.6, 402.9)	**412.1 (383.4, 440.8)* (SMD = 0.53, p = 0.008)**	365.7 (337.6, 393.9)	383.1 (354.8, 411.5)	**0.036**	**5.75 (0.23, 11.27)**	**0.041**	0.56 (-0.52, 1.63)	0.310
**Sleep Bouts, number**	78.0 (72.7, 83.2)	81.1 (75.8, 86.4)	72.4 (67.1, 77.6)	73.1 (67.8, 78.4)	**64.6 (59.2, 69.9)* (SMD = -0.75, p = 0.001)**	**69.1 (63.8, 74.4)* (SMD = -0.49, p = 0.022)**	77.0 (71.6, 82.3)	**0.0003**	**-1.24 (-2.27, -0.21)**	**0.019**	0.02 (-0.19, 0.22)	0.867
**Sleep Latency, minutes**	7.12 (5.99, 8.25)	8.11 (6.96, 9.26)	5.63 (4.50, 6.76)	7.12 (5.97, 8.27)	6.05 (4.90, 7.20)	6.44 (5.29, 7.59)	6.38 (5.22, 7.53)	0.064	-0.181 (-0.400, 0.038)	0.104	-0.020 (-0.063, 0.024)	0.378
**Bout Length, minutes**	5.18 (4.42, 5.95)	4.57 (3.80, 5.34)	5.49 (4.73, 6.25)	5.38 (4.61, 6.15)	**6.87 (6.10, 7.64)* (SMD = 0.54, p = 0.003)**	6.13 (5.36, 6.90)	5.53 (4.76, 6.29)	**0.003**	**0.195 (0.047, 0.343)**	**0.010**	0.012 (-0.017, 0.042)	0.418

†Model estimated mean and 95% confidence interval, adjusted for replicate and baseline values of phenotype; ‡p-value from ANOVA testing whether there are any differences in phenotype among dosage groups; §Results from linear model treating dose as an ordinal variable – β represents the expected change in phenotype associated with increasing to the next highest dosage group; ¶Results from continuous dosage model – β represents the expected change in phenotype for 1 µM increase in dose;*p<0.05 compared to DMSO in pairwise comparisons (performed only when ANOVA p<0.05). SMD and pairwise comparison results are indicated only for the sleep traits that displayed ANOVA p<0.05.

Table S10. Sleep of Zebrafish Larvae During the Lights Off Period Following the Daytime Melatonin Administration as a Positive Control in ZD7288 Screening Assay

Phenotype	Adjusted Mean (95% CI)†		Difference (95% CI)‡	p§
	DMSO	Melatonin		
**Total Sleep, minutes**	348.3 (318.0, 378.5)	337.5 (307.2, 367.7)	-10.8 (-54.3, 32.7)	0.621
**Sleep Bouts, number**	80.5 (74.6, 86.4)	78.2 (72.2, 84.1)	-2.3 (-10.7, 6.1)	0.582
**Sleep Latency, minutes**	7.05 (5.74, 8.36)	7.38 (6.07, 8.69)	0.33 (-1.53, 2.19)	0.728
**Bout Length, minutes**	4.56 (3.90, 5.22)	4.59 (3.93, 5.26)	0.04 (-0.90, 0.97)	0.939

†Model estimated mean and 95% confidence interval, adjusted for replicate and baseline values of phenotype; ‡Adjusted mean difference and 95% CI between melatonin and DMSO groups; §p-value comparing phenotype between DMSO and Melatonin groups.
